# Strong, conductive carbon nanotube fibers as efficient hole collectors

**DOI:** 10.1186/1556-276X-7-137

**Published:** 2012-02-17

**Authors:** Yi Jia, Xiao Li, Peixu Li, Kunlin Wang, Anyuan Cao, Jinquan Wei, Hongwei Zhu, Dehai Wu

**Affiliations:** 1 Key Laboratory for Advanced Materials Processing Technology, Ministry of Education and Department of Mechanical Engineering, Tsinghua University, Beijing, 100084, People's Republic of China; 2 Department of Advanced Materials and Nanotechnology, College of Engineering, Peking University, Beijing, 100871, People's Republic of China; 3 Center for Nano and Micro Mechanics, Tsinghua University, Beijing, 100084, People's Republic of China

**Keywords:** carbon nanotubes, fibers, heterojunction, solar cells

## Abstract

We present the photovoltaic properties of heterojunctions made from single-walled carbon nanotube (SWNT) fibers and n-type silicon wafers. The use of the opaque SWNT fiber allows photo-generated holes to transport along the axis direction of the fiber. The heterojunction solar cells show conversion efficiencies of up to 3.1% (actual) and 10.6% (nominal) at AM1.5 condition. In addition, the use of strong, environmentally benign carbon nanotube fibers provides excellent structural stability of the photovoltaic devices.

## Introduction

As a symbolic nanomaterial, carbon nanotube (CNT) with unique properties like high strength, high electrical conductivity, and chemical inertness has found important applications in optoelectronics [1], being an ideal candidate for various components in photovoltaic devices [2]. CNT bundles can be organized into two typical macrostructures: fibers (1D) and films (2D). The fabrication of homogeneous CNT films with a controllable thickness has been an important basis for the research on CNT-involved devices where CNTs mainly function as transparent electrodes [3]. Our recent work on CNT/Si heterojunction solar cells [4,5] have stimulated a series of studies on the photovoltaic properties of various heterostructures, including CNT/Si [6-16], CNT/CdTe [17], and graphene/Si Schottky junctions [18,19]. Among these devices, the CNT film serves multiple functions as a hole collector, charge transport path, and transparent electrode. However, the CNT film composed of CNT networks has a lot of inter-bundle voids, which should be fairly controlled to achieve high transparency while maintaining sufficient lateral conductivity of the film. The junction resistances between tubes/bundles also yield a limiting value for the conductivities for CNT films [20].

The CNT fiber is yet another macroscopic assembly of CNT bundles in a densified manner. CNT fibers have attracted intensive experimental and theoretical interests and are of increasing practical importance because of their unique 1D structure inherited from individual CNTs [21]. Early research efforts mainly focused on organizing discontinuous nanotubes into ribbon/fiber-like materials. We first reported that long single-walled CNT (SWNT) strands consisting of aligned SWNTs could be synthesized directly with a vertical floating chemical vapor deposition (CVD) method [22]. Many approaches have been developed since then for the assembly of CNTs into continuous fibers through direct spinning [23-26] and post-synthesis spinning [27-30]. Compared to the CNT film, the 1D CNT fiber composed of densely aligned CNT bundles has higher conductance. When forming a heterojunction with silicon, though the fiber itself (generally microns thick) is essentially opaque, the photo-generated charge holes excited from the exposed underlying silicon wafer will transport to it.

The purposes of this work are to introduce the design of the heterojunction solar cells using SWNT fibers as upper electrodes and n-type silicon wafers (n-Si) as photoactive electrodes and to investigate experimentally the photovoltaic properties of the SWNT fiber/Si heterojunctions, verifying the role of SWNTs as hole collectors.

## Experiment

**Figure 1:**
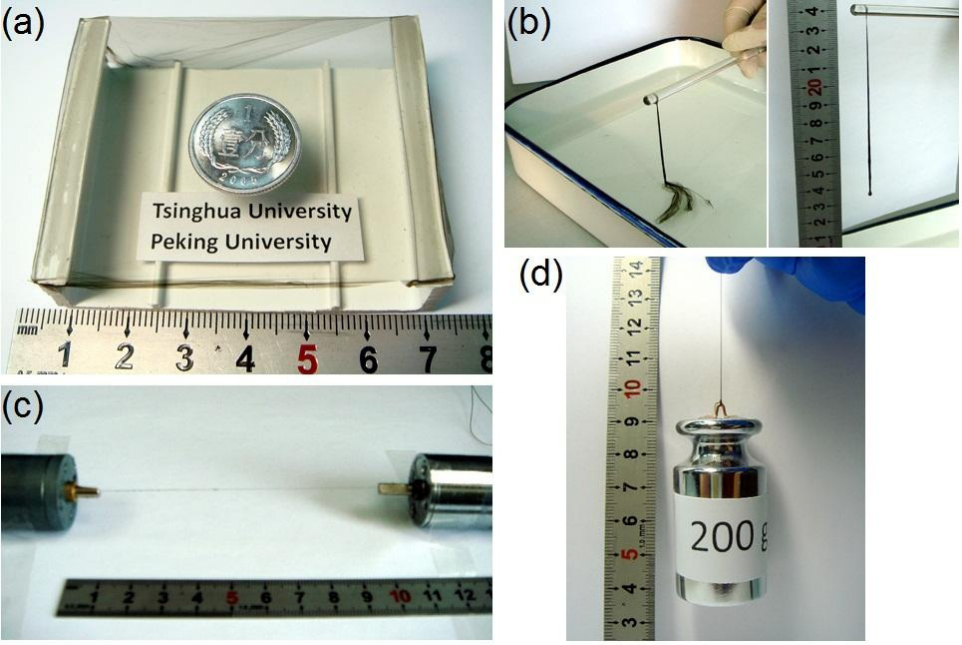
**Film-to-fiber processing**. (**a**) Freestanding SWNT thin film with a coin on it. (**b**) Fiber formation through a wetting/drying process. (**c**) Fiber twisting. (**d**) A single fiber bearing a 200-g weight.

The SWNT fibers used in this study were obtained by a simple film-to-fiber processing reported previously by our group [31]. SWNT films were first prepared by a floating CVD technique with a liquid precursor: a solution of xylene, ferrocene (0.36 mol/L), and sulfur (0.036 mol/L) [32]. Figure 1a shows the as-grown SWNT film hung over a ceramic tray. The film is stiff enough to bear a one-cent-coin weight. The freestanding film is highly transparent and continuous with a large area of approximately 50 cm^2^; the letters behind can be clearly seen through the film. Highly pure (> 98%) SWNT thin films were then obtained by a two-step posttreatment: hydrogen peroxide oxidation by immersing the films in 30% H_2_O_2 _solution for 72 h and then rinsing with hydrochloric acid (37% HCl) to remove amorphous impurities and iron catalyst. Smooth and homogenous films could be obtained when ethanol was dropped on the purified samples. A Langmuir monolayer of SWNTs was formed during the spreading of the ethanol layer along the water surface. The film was then picked up slowly with a glass rod (Figure 1b) and allowed to be further densified into a fiber upon drying. As shown in Figure 1c, the fiber was then twisted under stretching using two motors for 5 to approximately 10 min with a rotating speed of 30 rpm to improve its bulk density and the alignment of the SWNT bundles.

## Results and discussion

**Figure 2:**
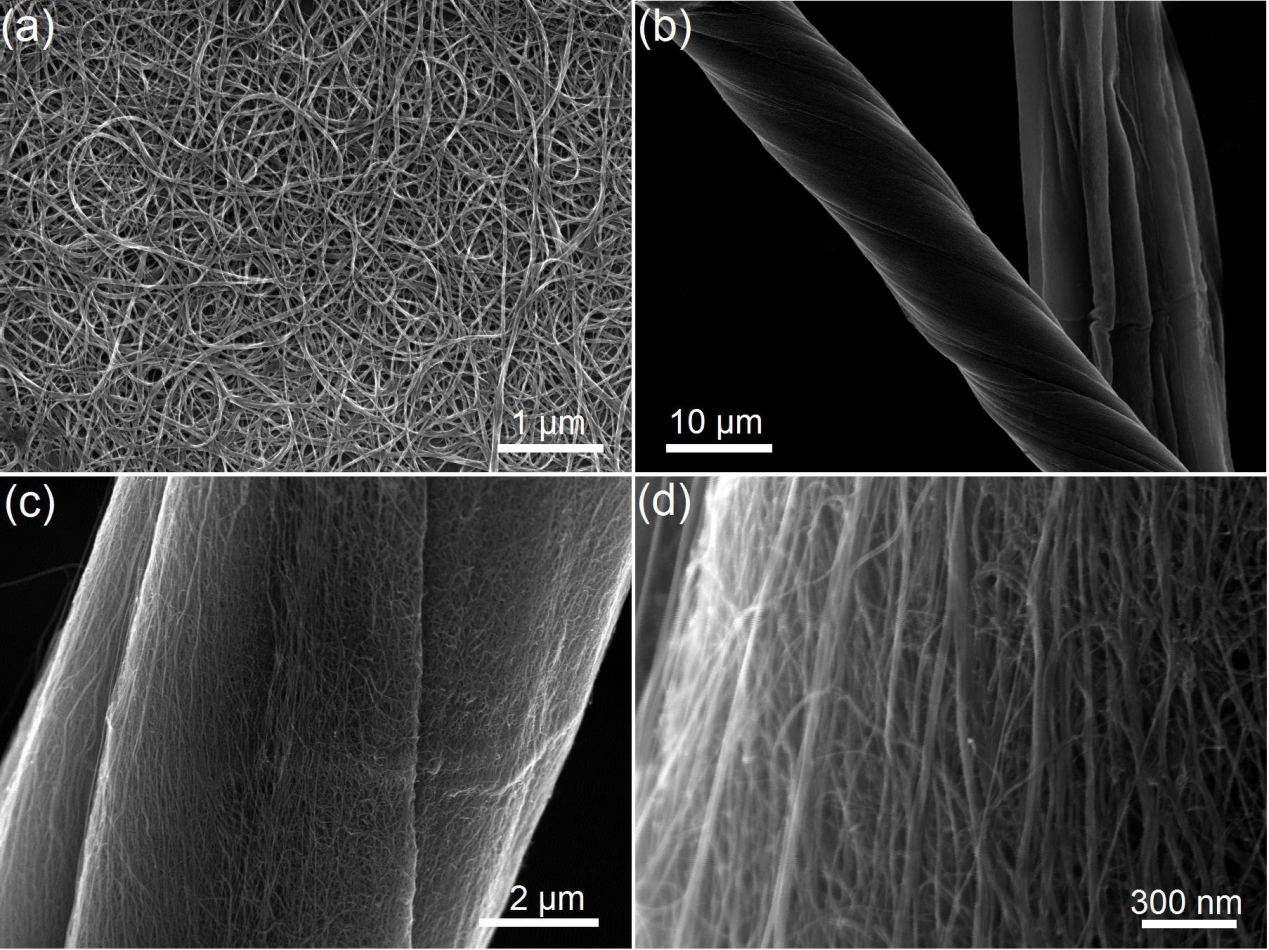
**SEM images**. (**a**) The single-walled CNT (SWNT) film and (**b, c, d**) a densified and twisted SWNT fiber.

A scanning electron microscope (SEM) image (Figure 2a) of the SWNT film reveals uniformity of the film across the entire area. Upon twisting, the SWNT fiber became stronger and tougher thanks to the closer contact and improved load transfer between nanotubes due to the enhanced van der Waals forces and friction, which is consistent with previously reported results [27,29,30]. Figure 1d illustrates the strength of a twisted SWNT fiber which sustains a 200-g weight. As further revealed by Figure 2b, d, the SWNT fiber upon twisting became much denser and possessed substantial alignment of the nanotubes along the twisting direction. The fiber diameter was reduced by approximately 35% from 17 to 11 μm. The twist angle, defined as the angle between the longitudinal direction of the SWNT bundles and the axis of the fiber, is about 26°, which is large enough to yield a strong fiber [29]. The result shows that this simple process allowed one-step formation of continuous nanotube fibers.

**Figure 3:**
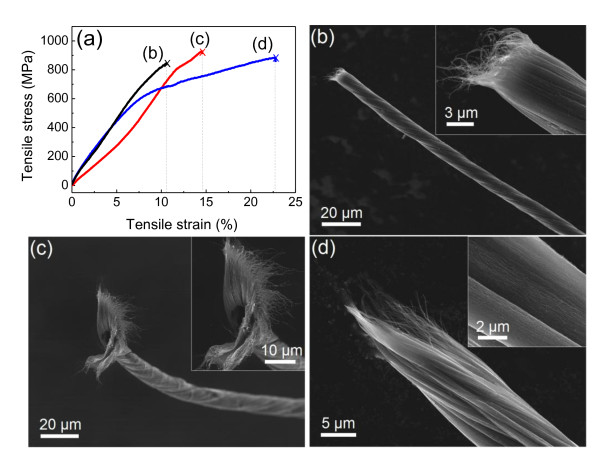
**Mechanical properties of the single-walled CNT (SWNT) fibers**. (**a**) Tensile stress-strain curves of three SWNT fibers. (**b**, **c**, **d**) SEM images of fractured SWNT fibers.

Before solar cell assembly, the mechanical properties of the SWNT fibers are tested. Figure 3a shows typical stress-strain curves for three SWNT fibers before fracture. All the SWNT fibers fractured at the highest load. The tensile strength and Young's modulus of our SWNT fibers were measured in the range 0.8 to 1.0 GPa and 8 to 10 GPa, respectively. During loading to failure, the fibers, and hence the SWNT bundles, experienced two different strains, elastic strain and plastic strain, owing to slippage between aligned bundles and plastic deformation of individual nanotubes. Three different fracture morphologies were observed: (1) brittle fracture due to strong inter-bundle coupling (Figure 3b), (2) fan-shaped fracture surface due to fiber unwinding (Figure 3c), and (3) sliding of bundles due to weak inter-bundle coupling and small twist angle (approximately 11°) (Figure 3d).

**Figure 4:**
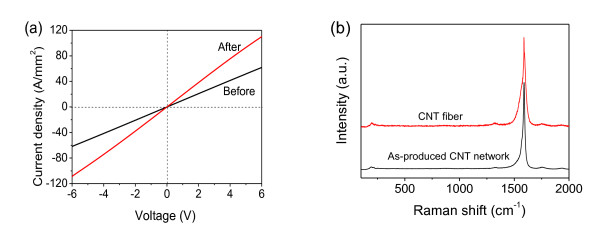
**Conducting properties of the single-walled CNT (SWNT) fibers**. (**a**)Current density-voltage curves of a SWNT fiber before and after twisting. (**b**) Raman spectra of as-produced CNT network and CNT fiber.

The high tensile strengths of the SWNT fibers are consistent with their electrical conducting performance. Owing to the higher density, the conducting properties of the twisted fibers are superior to the original fibers. Figure 4a shows the current density versus voltage curves of a typical SWNT fiber (approximately 1 cm long) before and after twisting. The current density is defined as the current per unit cross-sectional area of the SWNT fiber. The conductivity was enhanced featured with the resistivity reduced by approximately 40% from 9.7 × 10^-4 ^to 5.5 × 10^-4 ^Ω∙cm^-1^. Raman spectra at an excitation of 633 nm show high G-band intensity (*I*_G_) and very low D-band intensity (*I*_D_) of as-produced CNT network (black) and CNT fiber (red) in Figure 4b. The ratios of *I*_G_/*I*_D _are about 30, indicating high crystallization of CNT and negligible amorphous carbon. The two peak positions remain unchanged (D-band at 1,322 cm^-1 ^and G-band at 1,589 cm^-1^), revealing an absence of optical absorption change during the fiber twisting process.

**Figure 5:**
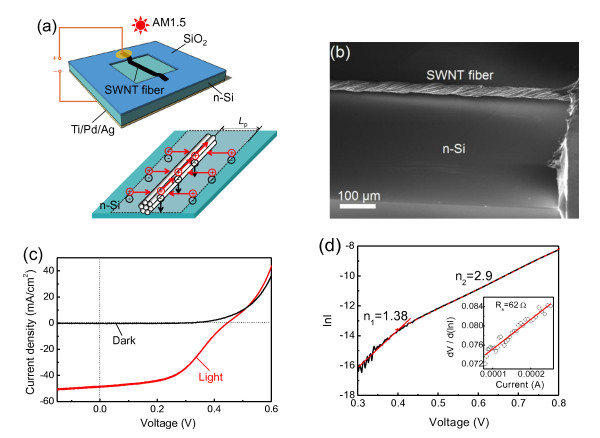
**The single-walled CNT (SWNT) fiber/n-Si solar cell**. (**a**) Device schematics of the SWNT fiber/n-Si solar cell. (**b**) SEM image of the SWNT fiber/n-Si junction. (**c**) Dark and light (AM1.5) *J*-*V *curves of the SWNT fiber/n-Si solar cell. (**d**) ln*I*-*V *plot and (inset) d*V*/d(ln*I*)-*I *plot.

Because the SWNT fibers were of macroscopic lengths and provided 1D electrical conducting channels, photovoltaic tests have been performed on the heterojunction solar cells made from the fibers and n-Si. The SWNT fiber/n-Si heterojunction was fabricated as illustrated in Figure 5a. An n-type Si (100) wafer (doping density, 2 × 10^15 ^cm^-3^) with a 300-nm SiO_2 _layer was patterned by photolithography and wet-etching to make a square window of 9 mm^2^. A back electrode of a Ti/Pd/Ag layer was used to ensure high-quality Ohm contact with the silicon. A SWNT fiber was then transferred to the top of the patterned silicon wafer and naturally dried. To introduce a strong adhesion between the fiber and the wafer, a piece of transparent tape was coated on the fiber. Forward bias was defined as positive voltage applied to the SWNT fiber. The current-voltage data were recorded using a Keithley 2601 SourceMeter (Keithley Instruments, Inc., Cleveland, OH, USA). The solar devices were tested with a Newport solar simulator (Newport, Beijing, China) under AM1.5 condition.

As illustrated in the bottom panel of Figure 5a, the fiber acted as a hole collector to extract the photo-excited holes generated within the rectangle region (marked with a dashed line) defined by the minority diffusion length (*L*_p_) (approximately 20 μm for n-Si at 2 × 10^15 ^cm^-3 ^doping level) of the silicon and the fiber length. Figure 5b shows a SEM image of the SWNT fiber/n-Si junction.

Figure 5c shows the measured current density-voltage (*J*-*V*) characteristics for a typical SWNT fiber/Si cell. Based on the *J*-*V *characteristics, the energy conversion efficiency (*η*) of the solar cell was estimated. The efficiency is defined by

$$\eta =j_{\mathrm{sc}}\cdot V_{\mathrm{oc}}\cdot \text{FF}/P_{\text{in}}$$

where *J*_sc _is the short-circuit current density (*J*_sc _= *I_sc _*/*S*). Here, the nominal current density is defined as the current per unit projectional area (*S*_n _= length × diameter) of the SWNT fiber; the actual current density is defined as the current per unit area when the minority diffusion in silicon is considered (*S*_a _= *S*_n _+ 2*L*_p _× length). Correspondingly, the actual efficiency (*η*_a_) and nominal efficiency (*η*_n_) will be obtained. *V*_oc _is the open-circuit voltage, *P*_in _is the incident power density (100 mW/cm^2^), and FF is the fill factor, which is defined by the relation

$$\text{FF}=J_{m}\cdot V_{m}/J_{\mathrm{sc}}\cdot V_{\text{oc}}$$

where (*J*_m_*V*_m_) is the maximum power point of the *J*-*V *characteristic of the solar cell.

Along with the other two tested cells, the photovoltaic performance of the three cells is summarized in Table 1. Initial tests have shown *η*_a _of 2% to approximately 3% and *η*_n _of 6% to approximately 10% at AM1.5, proving that SWNT fiber-on-Si is a potentially suitable configuration for making solar cells. Comparing sample #1 and sample #2 with different diameters in Table 1 the smaller diameter results in a smaller projectional area (*S*_n_) and entire effective area (*S*_a_), leading to a higher cell efficiency.

**Table 1:** Photovoltaic performance of the three SWNT fiber/n-Si solar cells.

Samples	Diameter	*S*_a_	*S*_n_	*I*_sc_	*V*_oc_	FF	*η*_a_	*η*_n_
	(μm)	(10^-4 ^cm^2^)	(10^-4 ^cm^2^)	(μA)	(V)	(%)	(%)	(%)
#1	17.1	17.1	5.13	24.9	0.445	49.1	3.17	10.6
#2	29.8	21.1	8.95	33.8	0.475	40.1	3.07	7.19
#3	18.6	17.6	5.57	22.1	0.414	41.4	2.16	6.80

*S*_a_, actual current density; *S*_n_, nominal current density; *I*_sc_, short-circuit current; FF, fill factor; *η*_a_, actual efficiency; *η*_n_, nominal efficiency; *V*_oc_, open-circuit voltage.

As shown in Figure 5c, the *V*_oc _and FF of the SWNT fiber/Si device are 0.445 V and 49.1%, respectively, which are comparable to the values for CNT film/Si cells [32]. The overall *η*_n _of the fiber device (approximately 10.6%) is about 43% higher than that of the film device (approximately 7.4%). This disparity arose mainly from the different definition of the junction area for these two devices. In this fiber device, the *η*_a _is 3.17% when the entire effective area is used instead of only the fiber projection area. It is worth mentioning that the size of the inter-bundle voids within a CNT film is < 5 μm [32], which is substantially smaller than the *L*_p _(20 μm). This implies that the SWNT bundles with an inter-spacing of 2 *L*_p _will give the optimal charge collection. The cell efficiencies are expected to be further improved by acid doping [16].

Consistent with the characteristics of the 1D/2D junction, we note that the device only shows a moderate rectification ratio which is approximately 1,680 at ± 0.8 V, and a typical reverse current at -1.0 V is 250 nA. As shown in Figure 5d, at low forward voltages, the current follows an exponential dependence with ideality factor (*n*) equal to 1.38. At higher voltages, the current follows an exponential dependence with an ideality factor of 2.9. This variation corresponds to a transition between two regimes [33]: (1) the current is dominated by diffusion and generation-recombination outside the space charge region (*n *= 1), and (2) the high-injection regime, where the density of the minority carrier is comparable with that of the majority (*n *= 2). A d*V*/d(ln*I*)-*I *plot (Figure 5d, inset) is used to analyze the current-voltage characteristics when the series resistance (*R*_s_) begins to dominate, yielding a *R*_s _of approximately 62 Ω.

The 1D nature of the SWNT fiber offers a tremendous opportunity for exciton dissociation. SWNTs in the devices are involved in multiple processes including hole collecting and transporting. Despite its opaque feature and the relatively small interfacial area for charge separation, the SWNT fiber provides many 1D paths, forming a conducting channel for charge transport.

The devices present a great potential for use as photovoltaic solar cells and light sensors. In addition to enhancing photovoltaic conversion efficiency, the incorporation of the robust SWNT fibers can potentially improve the mechanical and environmental stability of the devices.

## Conclusions

To conclude, we have demonstrated the photovoltaic properties of the SWNT fiber/Si heterojunction and revealed that SWNTs can be used as efficient hole collectors. The SWNT fiber/n-Si solar cell studied here represents an addition to the CNT film/n-Si counterparts reported by us previously. The photovoltaic devices also show excellent structural stability due to the use of strong, environmentally benign CNT fibers.

## Competing interests

The authors declare that they have no competing interests.

## Authors' contributions

YJ carried out the solar cell assembly and test, and drafted the manuscript. XL participated in the solar cell assembly. PL prepared the carbon nanotube films. AC, DW, and HZ conceived of the study and participated in its design and coordination. JW and KW participated in the data analysis. All authors read and approved the final manuscript.

## References

[B1] Avouris P Freitag M Perebeinos V Carbon-nanotube photonics and optoelectronics Nat Photon 2008 2 341 350 10.1038/nphoton.2008.94

[B2] Zhu HW Wei JQ Wang KL Wu DH Applications of carbon materials in photovoltaic solar cells Sol Energy Mater Sol Cells 2009 93 1461 1470 10.1016/j.solmat.2009.04.006

[B3] Zhu HW Wei BQ Assembly and applications of carbon nanotube thin films J Mater Sci Tech 2008 24 447 456

[B4] Wei JQ Jia Y Shu QK Gu ZY Wang KL Zhuang DM Zhang G Wang ZC Luo JB Cao AY Wu DH Double-walled carbon nanotube solar cells Nano Lett 2007 7 2317 2321 10.1021/nl070961c 17608444

[B5] Jia Y Wei JQ Wang KL Cao AY Shu QK Gui XC Zhu YQ Zhuang DM Zhang G Ma BB Wang LD Liu WJ Wang ZC Luo JB Wu DH Nanotube-silicon heterojunction solar cells Adv Mater 2008 20 4594 4598 10.1002/adma.200801810

[B6] Zhou H Colli A Ahnood A Yang Y Rupesinghe N Butler T Haneef I Hiralal P Nathan A Amaratunga GAJ Arrays of parallel connected coaxial multiwall carbon nanotube amorphous silicon solar cells Adv Mater 2009 21 3919 3923 10.1002/adma.200901094

[B7] Arena A Donato N Saitta G Galvagno S Milone C Pistone A Photovoltaic properties of multi-walled carbon nanotubes deposited on n-doped silicon Microelectronics J 2008 39 1659 1662 10.1016/j.mejo.2008.02.012

[B8] Li ZR Kunets VP Saini V Xu Y Dervishi E Salamo GJ Biris AR Biris AS SOCl_2 _enhanced photovoltaic conversion of single wall carbon nanotube/n-silicon heterojunctions Appl Phys Lett 2008 93 243117 10.1063/1.3050465

[B9] Li ZR Kunets VP Saini V Xu Y Dervishi E Salamo GJ Biris AS Light-harvesting using high density p-type single wall carbon nanotube/n-type silicon heterojunctions ACS Nano 2009 3 1407 1414 10.1021/nn900197h 19456166

[B10] Ong PL Euler WB Levitsky IA Hybrid solar cells based on single-walled carbon nanotubes/Si heterojunctions Nanotechnol 2010 21 105203 10.1088/0957-4484/21/10/105203 20157233

[B11] Li CY Li Z Zhu HW Wang KL Wei JQ Li X Sun PZ Zhang H Wu DH Graphene nano-"patches" on carbon nanotube network for highly transparent/conductive thin film applications J Phys Chem C 2010 114 14008 14012 10.1021/jp1041487

[B12] Jia Y Li PX Wei JQ Cao AY Wang KL Li CL Zhuang DM Zhu HW Wu DH Carbon nanotube films by filtration for nanotube-silicon heterojunction solar cells Mater Res Bull 2010 45 1401 1405 10.1016/j.materresbull.2010.06.045

[B13] Shu QK Wei JQ Wang KL Zhu HW Li Z Jia Y Gui XC Guo N Li XM Ma CR Wu DH Hybrid heterojunction and photoelectrochemistry solar cell based on silicon nanowires and double-walled carbon nanotubes Nano Lett 2009 9 4338 4342 10.1021/nl902581k 19852483

[B14] Shu QK Wei JQ Wang KL Song S Guo N Jia Y Li Z Xu Y Cao AY Zhu HW Wu DH Efficient energy conversion of nanotube/nanowire-based solar cells Chem Commun 2010 46 5533 5535 10.1039/c0cc00512f 20577694

[B15] Jia Y Cao AY Bai X Li Z Zhang LH Guo N Wei JQ Wang KL Zhu HW Wu DH Achieving high efficiency silicon-carbon nanotube heterojunction solar cells by acid doping Nano Lett 2011 11 1901 1905 10.1021/nl2002632 21452837

[B16] Jia Y Cao AY Li PX Gui XC Zhang LH Wei JQ Wang KL Zhu HW Xu Y Wu DH Encapsulated carbon nanotube-oxide-silicon solar cells with stable 10% efficiency Appl Phys Lett 2011 98 133115 10.1063/1.3573829

[B17] Zhang LH Jia Y Wang SS Li Z Ji CY Wei JQ Zhu HW Wang KL Wu DH Shi EZ Fang Y Cao AY Carbon nanotube and CdSe nanobelt Schottky junction solar cells Nano Lett 2010 10 3583 3589 10.1021/nl101888y 20715803

[B18] Li XM Zhu HW Wang KL Cao AY Wei JQ Li CY Jia Y Li Z Li X Wu DH Graphene-on-silicon Schottky junction solar cells Adv Mater 2010 22 2743 2748 10.1002/adma.200904383 20379996

[B19] Li X Li CY Zhu HW Wang KL Wei JQ Li XM Xu EY Li Z Luo S Lei Y Wu DH Hybrid thin films of graphene nanowhiskers and amorphous carbon as transparent conductors Chem Commun 2010 46 3502 3504 10.1039/c002092c 20376392

[B20] Pereira LFC Rocha CG Latgé A Coleman JN Ferreira MS Upper bound for the conductivity of nanotube networks Appl Phys Lett 2009 95 123106 10.1063/1.3236534

[B21] Behabtu N Green MJ Pasqualia M Carbon nanotube-based neat fibers Nanotoday 2008 3 24 34

[B22] Zhu HW Xu CL Wu DH Wei BQ Vajtai R Ajayan PM Direct synthesis of long single-walled carbon nanotube strands Science 2002 296 884 886 10.1126/science.1066996 11988567

[B23] Li YL Kinloch IA Windle AH Direct spinning of carbon nanotube fibers from chemical vapor deposition synthesis Science 2004 304 276 278 10.1126/science.1094982 15016960

[B24] Motta M Moisala A Kinloch IA Windle AH High performance fibres from 'dog bone' carbon nanotubes Adv Mater 2007 19 3721 3726 10.1002/adma.200700516

[B25] Koziol K Vilatela J Moisala A Motta M Cunniff P Sennett M Windle A High-performance carbon nanotube fiber Science 2007 318 1892 1895 10.1126/science.1147635 18006708

[B26] Vilatela JJ Windle AH Yarn-like carbon nanotube fibers Adv Mater in press doi: 10.1002/adma.201002131 10.1002/adma.201002131 20809514

[B27] Zhang M Atkinson KR Baughman RH Multifunctional carbon nanotube yarns by downsizing an ancient technology Science 2004 306 1358 1361 10.1126/science.1104276 15550667

[B28] Ericson LM Fan H Peng H Davis VA Zhou W Sulpizio J Wang YH Booker R Vavro J Guthy C Parra-Vasquez ANG Kim MJ Ramesh S Saini R Kittrell C Lavin G Schimdt H Adams WW Billups WE Pasquali M Hwang WH Hauge RH Fischer JE Smalley RE Macroscopic, neat, single-walled carbon nanotube fibers Science 2004 305 1447 1450 10.1126/science.1101398 15353797

[B29] Zhang XF Li QW Tu Y Li Y Coulter JY Zheng LX Zhao YH Jia QX Peterson DE Zhu YT Strong carbon-nanotube fibers spun from long carbon-nanotube arrays Small 2007 3 244 248 10.1002/smll.200600368 17262764

[B30] Zhang XF Li QW Holesinger TG Arendt PN Huang JY Kirven PD Clapp TG DePaula RF Liao XZ Zhao YH Zheng LX Peterson DE Zhu YT Ultrastrong, stiff, and lightweight carbon-nanotube fibers Adv Mater 2007 19 4198 4201 10.1002/adma.200700776

[B31] Li X Li CY Li XM Zhu HW Wei JQ Wang KL Wu DH Force- and light-controlled electrical transport characteristics of carbon nanotube bulk junctions Chem Phys Lett 2009 481 224 228 10.1016/j.cplett.2009.09.097

[B32] Li Z Jia Y Wei JQ Wang KL Shu QK Gui XC Zhu HW Cao AY Wu DH Large area, highly transparent carbon nanotube spiderwebs for energy harvesting J Mater Chem 2010 20 7236 7240 10.1039/c0jm01361g

[B33] Sze SM Ng KK The Physics of Semiconductor Devices 2007 3 New York: Wiley Interscience

